# Transforming growth factor beta isoforms regulation of Akt activity and XIAP levels in rat endometrium during estrous cycle, in a model of pseudopregnancy and in cultured decidual cells

**DOI:** 10.1186/1477-7827-7-80

**Published:** 2009-08-05

**Authors:** Pierre-Luc Caron, Guylaine Fréchette-Frigon, Carl Shooner, Valérie Leblanc, Eric Asselin

**Affiliations:** 1 Départment de Chimie-Biologie, Groupe de Recherche en Biopathologies Cellulaires et Moléculaires, Université du Québec à Trois-Rivières, C.P. 500, Trois-Rivières, Québec, G9A 5H7, Canada

## Abstract

**Background:**

During the estrous cycle, the rat uterine endometrium undergoes many changes such as cell proliferation and apoptosis. If implantation occurs, stromal cells differentiate into decidual cells and near the end of pregnancy, a second wave of apoptosis occurs. This process called decidual regression, is tightly regulated as is it crucial for successful pregnancy. We have previously shown that TGF-beta1, TGF-beta2 and TGF-beta3 are expressed in the endometrium during decidual basalis regression, but although we had demonstrated that TGF- beta1 was involved in the regulation of apoptosis in decidual cells, the ability of TGF- beta2 and TGF-beta3 isoforms to trigger apoptotic mechanisms in these cells remains unknown. Moreover, we hypothesized that the TGF-betas were also present and regulated in the non-pregnant endometrium during the estrous cycle. The aim of the present study was to determine and compare the specific effect of each TGF-β isoform in the regulation of apoptosis in sensitized endometrial stromal cells in vitro, and to investigate the regulation of TGF-beta isoforms in the endometrium during the estrous cycle in vivo.

**Methods:**

Rats with regular estrous cycle (4 days) were killed at different days of estrous cycle (diestrus, proestrus, estrus and metestrus). Pseudopregnancy was induced with sex steroids in ovariectomized rats and rats were killed at different days (days 1–9). Uteri were collected and either fixed for immunohistochemical staining (IHC) or processed for RT-PCR and Western analyses. For the in vitro part of the study, rats were ovariectomized and decidualization was induced using sex steroids. Endometrial stromal decidual cells were purified, cultured and treated with different concentrations of TGF-beta isoforms.

**Results:**

Our results showed that all three TGF-beta isoforms are present, but are localized differently in the endometrium during the estrous cycle and their expression is regulated differently during pseudopregnancy. In cultured stromal cells, we found that TGF-beta3 isoform induced Smad2 phosphorylation, indicating that the Smad pathway is activated by TGF-beta3 in these cells. Furthermore, TGF-beta2 and TGF-beta3 induced a dose-dependant increase of apoptosis in cultured stromal cells, as demonstrated by Hoechst nuclear staining. Noteworthy, TGF-beta2 and TGF-beta3 reduced the level of the anti-apoptotic XIAP protein, as well as the level of phosphorylated/active Akt, a well known survival protein, in a dose-dependent manner.

**Conclusion:**

Those results suggest that TGF-beta might play an important role in the remodelling endometrium during the estrous cycle and in the regulation of apoptosis in rat decidual cells, in which inhibition of Akt survival pathway might be an important mechanism involved in the regulation of apoptosis.

## Background

During the pre-implantation period, the uterine endometrium undergoes morphological and histological changes including cell proliferation, differentiation and apoptosis to provide the best environment for embryo implantation. Sex steroids, estrogen and progesterone, are responsible for these changes during the estrous cycle. Estrogen stimulate cell proliferation whereas progesterone inhibits it [1,2]. Luminal and glandular epithelium as well as stromal cells proliferate and degenerate in response to cyclic changes in serum steroids hormones [3,4]. In absence of embryonic factors, endometrial cells undergo apoptosis or programmed cell death. However, when embryo implantation occurs, many changes begin in the endometrium in which stromal cells proliferate in response to estrogen and progesterone to form the decidual cells, a process called decidualization. [5,6]. Near the end of the pregnancy, on day 14, decidual cells regress by apoptosis, a phenomenon called decidual regression [5,7,8]. Our group and others have shown apoptosis in the luminal epithelium at estrus in mice [4] and rats [9-11] during the estrous cycle; apoptosis was also found in stromal cells at diestrus in mice [4]. We have previously shown that Akt, an important kinase involved in the control of endometrial cell proliferation, was decreased at estrus and was accompanied by an increase in apoptosis in luminal epithelial cells [10]. Studies have shown that apoptosis is increased in the rat endometrium during the decidual regression [5,12,13]. Although changes in uterine endometrium are regulated by steroids hormones [4], they are also regulated by growth factors, such as transforming growth factor-β (TGF-β).

Transforming growth factor-β was originally identified by its ability to induce a transformed phenotype in normal rat kidney fibroblasts in culture [14-16]. TGF-βs are now known to be multifunctional proteins involved in many biological processes such as cell proliferation and differentiation, tissue remodelling, angiogenesis, immunoregulation and regulation of extracellular matrix [17-20]. They are also inhibitors of cellular growth in many cell types [21]. It has been shown that they induce apoptosis [8] and it might be through the caspases pathway [22,23]. Three TGF-β isoforms are found in mammalians and their molecular weights are slightly different: TGF-β1 (15 kDa), TGF-β2 (12.5 kDa) and TGF-β3 (12 kDa). They share 80% sequence homology and genes encoding these isoforms are located on different chromosomes (chromosomes 19, 1 and 14 for TGF-β1, TGF-β2 and TGF-β3 respectively) [17,19,24]. TGF-β are produced in an inactive form which is activated by proteolytic cleavage [25]. Bioactive TGF-β signals through transmembrane serine/threonine kinase receptors to phosphorylate Smads proteins, the TGF-β intracellular effectors. Once activated, these proteins translocate to the nucleus, bind to DNA binding proteins and activate transcription of target genes [18,25]. Loss of TGF-β-mediated growth inhibition contributes to the development and progression of tumors [21,26]. The high degree of conservation of these isoforms among species and through evolution suggests an important regulation function [27]. Indeed, it has been shown that all three TGF-β isoforms are differently expressed during embryogenesis [20,28], in porcine [29,30] and bovine [31] conceptus-maternal interface and in the mouse uterus [32]. Moreover, we have shown in our laboratory that TGF-β isoforms efficiently inhibit cellular proliferation in human endometrial carcinoma cell lines and that TGF-β3 increases invasiveness of endometrial carcinoma cells through PI 3-K upregulation of XIAP and protein kinase C induction of MMP-9 [33].

Chegini *et al *showed by immunohistochemical analysis (IHC) that TGF-β1, TGF-β2 and TGF-β3 are expressed in the rat estrous cycle, but in a context of induced endometriosis [34]. Studies have also shown that apoptosis is increased during the decidual regression [5,12,13]. TGF-β1 and TGF-β2 mRNA are expressed in mouse uterus during pregnancy [35,36], and TGF-β1 and TGF-β2 have been shown to induce apoptosis in human [37] and rat cultured [8] endometrial stromal cells. The role of TGF-β3 in the process of decidual cell death has never been described before. Recently, we showed that TGF-β isoforms are differently localized and regulated in endometrial cells of pregnant rats during implantation and decidual regression, and that TGF-β1 is involved in the regulation of apoptosis in cultured decidual cells [38]. Although the expression of TGF-β isoforms have been described in uterus of pregnant rats, their expression during the estrous cycle and their effect on decidual cell regression remain to be explored.

The aim of this study was to investigate and characterize the expression of all three TGF-β isoforms in the rat uterus during the estrous cycle and to determine *in vitro *the specific effect of TGF-β2 and TGF-β3 on the regulation of survival of decidual cells. The current study is the first to show the expression and localization of all three TGF-β isoforms in the normal rat uterus and to provide evidence for the regulation of TGF-β2 and TGF-β3 by sex steroids using an artificially induced pseudopregnant rat model. Additionally, this study shows for the first time the regulation of Akt phosphorylation and XIAP expression in the process of decidual cell death induced by TGF-β2 and TGF-β3.

## Methods

### Reagents

TGF-β1 (sc-146, lot # F262, 200 μg/ml), TGF-β2 (sc-90, lot # B202, 200 μg/ml) and TGF-β3 (sc-82, lot # A222, 200 μg/ml) polyclonal antibodies were purchased from Santa Cruz Biotechnology, Inc (Santa Cruz, CA, USA). CDC47/MCM7 antibody was obtained from Medicorp (Montréal, QC, Canada). Phospho-Akt (Ser 473), Akt, XIAP, and Phospho-Smad2 (Ser 465/467) antibodies were obtained from Cell Signaling Technology (Beverly, MA, USA). Anti-Smad2 antibody was purchased from Zymed (San Francisco, CA, USA). β-actin antibody was purchased from Sigma-aldrich (St-Louis, Missouri, USA). Vectastain ABC Kit for rabbit IgG and NovaRed substrate were purchased from Vector Laboratories Inc. (Burlingame, CA, USA). Rabbit IgG was used at the same concentration as the primary antisera. Harris modified haematoxylin solution was obtained from Sigma-aldrich (St-Louis, Missouri, USA). TGF-β2 recombinant protein was purchased from Biosource (Cat # PHG9114, lot # 19698-04S, 5 μg, diluted at 50 μg/ml, QC, Canada) and TGF-β3 recombinant protein was purchased from Calbiochem (Cat # PF073, lot # D24438, 2 ug, diluted at 50 μg/ml, San Diego, CA, USA). 17β-estradiol (E2) was purchased from Sigma-aldrich (St-Louis, Missouri, USA) and progesterone (P4) from Laboratoire Mat (Québec, QC).

### Animals

Sprague-Dawley female rats, 200–225 g, were obtained from Charles River Laboratories Canada. Animals were maintained on standard chow and water, which were available *ad libitum*, in animal facilities illuminated between 6:00 h and 20:00 h. All procedures were performed in accordance with guidelines of the Canadian Council on Animal Care for the handling and training of laboratory animals and the Good Health and Animal Care Committee of the Université du Québec à Trois-Rivières. Stages of the estrous cycle were confirmed by vaginal smears. Rats with three regular cycles of four days were used in these experiments and killed at various stages of the estrous cycle (diestrus, proestrus, estrus, metestrus). Uteri were collected and fixed for immunohistochemical staining (IHC) or endometrial RNA and protein extracts collected by scraping the endometrium for RT-PCR and Western analysis. To induce pseudopregnancy, rats were ovariectomized for at least 10 days and then injected with E_2 _and P_4 _as described previously [39] 1) 0.2 μg estradiol injection per day for three days (in the morning, day -2, -1 and 0); 2) On the third day (day 0 of pseudopregnancy), another injection in the afternoon of estradiol (0.2 μg) and progesterone (1 mg) was performed; 3) No treatment for the next day; 4) Injections of progesterone (4 mg) for two days (day 2 and 3 of pseudopregnancy); 5) Injections of estradiol (0.3 μg) and progesterone (4 mg) for one day (day 4 of pseudopregnancy); 6) Injections of estradiol (0.1 μg) and progesterone (4 mg) for 5 days (days 5 to 9 of pseudopregnancy); 7) Rats were killed on day 1, 3, 5, 7 and 9 of pseudopregnancy. Three different rats were used for each time of pseudopregnancy. Endometrial protein extracts were collected by scraping the endometrium for Western analysis. E_2 _and P_4 _were dissolved with sesame oil, and administered by subcutaneous injection.

### Rat pretreatments and decidual endometrial stromal cell culture

A total of 10 rats were ovariectomized and then allowed to recover from surgery for a minimum of 10 days. They were pre-treated with physiological doses of estradiol (1,3,5(10)-Estratriene-3,17β-diol, Sigma-aldrich) and progesterone (Laboratoire Mat, PQ) to induce decidualization as described previously [40]: 1) 0.2 ug estradiol injection per day for three days (in the morning, day -2,-1 and 0); 2) On the third day (day 0 of pseudopregnancy), another injection in the afternoon of estradiol (0.2 μg) and progesterone (1 mg) was performed; 3) No treatment for 2 days (day 1 and 2 of pseudopregnancy); 4) Injections of estradiol (0.1 μg) and progesterone (4 mg) for three days (day 3, 4 and 5 of pseudopregnancy); 5) Another injection of estradiol (0.1 μg) in the afternoon on day 7 (day 4 of pseudopregnancy); 6) Rats were killed on day 8 (day 5 of pseudopregnancy). All endometrial stromal cells collected for cultures were recovered from rats treated with the protocol described above.

Uteri were removed and horns taken and immerged in HBSS solution containing HEPES (20 mM), penicillin (100 units/ml), streptomycin (100 μg/ml) and fungizone (1,25 μl/ml) (Invitrogen, ON, Canada). Further manipulations were performed in a sterile environment. The uterine horns were transferred into a sterile petri containing HBSS, slit longitudinally and immerse in trypsin type I solution (0.3%) (Roche Diagnostics, QC, Canada) in HBSS and agitated for 60 minutes at room temperature. Uterine horns were then vortexed at maximum for 5 sec and supernatant containing epithelial cells was discarded. Uterine horns were washed three times with 2.5 ml of HBSS and immersed in a HBSS solution containing trypsin type I (0.03%), DNAse I (0.016%) and collagenase type II (0.064%) for 15 minutes at 37°C in a water bath. Uterine horns were then vortexed at maximum for 5 sec. The supernatant containing stromal cells was transferred into a sterile falcon tube containing 150 μl of FBS D.C (Dextran-Charcoal extracted). Uterine horns were washed two times with 2.5 ml of HBSS and the supernatant was mixed with stromal cells. Uterine horns were discarded and stromal cells were centrifuged at 1000 g for 5 minutes. Cells were washed two times with HBSS and centrifuged. The supernatant was discarded and cells diluted with DMEM-F12 (pH 7.1) (Invitrogen, ON, Canada) containing 2.438 g/L NaHCO3, 10% FBS D.C. and gentamycine 50 μg/ml. Cells were incubated at 37°C in an atmosphere of 5% CO2. Cells were plated in 6-well plates (Corning plates) at a density of 50% (4 × 10^5 ^cells per well). The medium was changed two hours after the first incubation in order to eliminate epithelial cell contamination from stromal cell cultures. The purity of stromal cells was more than 97%: cell culture contamination with epithelial cells was evaluated by cellular morphology and immunofluorescence using a Keratin 8/18 antibody. Three to 5 days after plating (more than 90% of confluency reached), cells were treated for 24 hours in the presence or absence of increasing doses of TGF-β2 and TGF-β3 recombinant proteins. Total proteins from treated cell cultures were extracted using TRIZOL (Invitrogen, ON, Canada). For Western blot analyses, 10–15 μg of total protein per gel lane were used for each analysis.

### Immunohistochemical staining

The uterus was fixed in 4% paraformaldehyde solution and embedded in paraffin. Tissue sections 7 μm thick were mounted on polylysine-coated slides, deparaffinized, rehydrated, and then heated in 10 mM citrate buffer (pH 6) containing triton X-100 (Sigma-Aldrich) 0.1% (v/v). After two washes with PBS, slides were then incubated with 0.3% hydrogen peroxide in methanol for 30 min to quench endogenous peroxidase activity. After washing with PBS, tissues were incubated with blocking serum (Vectastain ABC Kit) at room temperature for 1 h. Then, a primary antibody diluted in blocking serum (TGF-β1, β2 or β3; 1:50 dilution) was added to the slides and incubated at 4°C overnight in a humidified chamber. After washing 5 min. in PBS, tissue sections were incubated for 30 min. with 3 μg/ml biotinylated antibody (anti-rabbit). Subsequently, slides were washed with PBS and incubated with avidin-biotin complex reagent containing horseradish peroxidase for 30 min. Slides were washed with PBS for 5 min and color development was achieved using NovaRed substrate. The tissue sections were counterstained with haematoxylin. Negative controls were performed using the same protocol but substituting the primary antibody with normal rabbit IgG (Vector Laboratories Inc., Burlingame, CA, USA).

### mRNA extraction and semi-quantitative RT-PCR analysis

Endometrium from each uterus of cycling rats was scraped using a glass microscope slide and homogenized using a pipette in TRIZOL (Invitrogen, ON, Canada). mRNA extraction was performed according to the manufacturer's instructions and diluted in RNAse-free DEPC water treated. In order to measure abundance of TGF-β1, TGF-β2, TGF-β3 mRNAs, primers were chosen as described below and tested with different primer concentrations. Total RNA (0.2 μg/μl) was used for preparation of first strand cDNA by reverse transcriptase (RT). The RNA samples were incubated (65°C, 10 min) with 2 ul oligo dT (deoxythymidine) primers in a final volume of 10 μl. Samples were then incubated (37°C, 60 min) in 20 μl of a reaction buffer (1×) containing dithiothreitol (DTT; 100 mM), deoxynucleotide triphosphates (dNTPs; 5 mM), and Muloney murine leukemia virus reverse transcriptase (MMLV-RT; 200 U/μl). After DNA synthesis, the reaction volumes were brought up to 60 μl with autoclaved water. A negative control was also included, using the same reaction mixture but without RNA to ensure absence of any contaminating genomic DNA in the RNA template. Rat TGF-β1 a 501 pb mRNA (572–1072 bp; Genbank accession number X52498) was amplified using sense primer 5'-CTGTCCAAACTAAGGCTCGC-3' and antisense primer 5'-ACTGAAGCGAAAGCCCTGTA-3'. For TGF-β2 mRNA, the expression was determined by amplification of 707 bp (143–849 bp; Genbank accession number NM_031131) and the sequence of the primers were 5'-ATCCTAGCCAGGGACGTTTT-3' (sense) and 5'-CGAAAGACCCTGAACTCTGC-3' (antisense). Expression of TGF-β3 mRNA was determined by amplification of 681 bp (663–1343 bp; Genbank accession number NM_013174). Amplification was carried out using the sense upstream sequence 5'GAAGGCTGCACTCAGGAAAC-3' and the antisense downstream sequence 5'-GCAGTTCTCCTCCAAGTTGC-3'. Rat β-actin mRNA was amplified using sense primer 5'-GAGGATCTTCATGAGGTAGTCTGTCAGGTC-3' and antisense primer 5'-CAACTGGAACGACATGGAGAAGATCTGGCA-3'. Each reaction mixture (final volume 50:1) contains RT template or negative control (5:1), MgCl_2 _(50 mM), primers (10 μM), and Taq DNA polymerase (5 U/μl). The PCR cycling conditions (35 cycles except for β-actin 25 cycles) chosen were 30 sec at 94°C, 30 sec at 58°C (TGF-β1 and TGF-β2) and 55°C (TGF-β3) and 30 sec (TGF-β1) and 1 min (TGF-β2 and TGF-β3) at 72°C, followed by a 5 min extension at 72°C. Reaction products were analyzed on 1.0% agarose gels. Bands were visualized by ethidium bromide staining. Densitometrical analyses were performed (mRNA of interest and β-actin) using the GelDoc 2000 and the Quantity One software (Bio-Rad, Mississauga, ON, Canada). Optimizations for all set of primers were performed prior analyses. All PCR reactions were analysed during the linear amplification phase. Results are expressed as a ratio (mRNA of interest/β-actin) to correct for loading for each endometrial sample.

### Protein extraction and Western analysis

Endometrium from each uterus of cycling and pseudopregnant rats was scraped using a glass microscope slide and homogenized using a pipette in TRIZOL (Invitrogen, ON, Canada). Protein extraction was achieved according to the manufacturer's instructions and diluted in 1% SDS. Homogenates were centrifuged to remove insoluble material. The supernatant was recovered and stored at -20°C pending analysis. Protein content was determined with the Bio-Rad DC Protein Assay. Protein extracts (50 μg) were heated at 94°C for 3 min, resolved by 12–15% SDS-PAGE and electrotransferred to nitrocellulose membranes using a semidry transfer (Bio-Rad, Mississauga, ON). The membranes were then blocked 2 h at room temperature with PBS containing 5% milk powder, then incubated with TGF-β1, TGF-β3 1:750; TGF-β2 1:2000; P-Smad2 (Ser 465/467) 1:1000; Smad2 1:1000; Akt 1:1000; Phospho-Akt 1:1000; XIAP 1:1500; CDC47/MCM7 1:1000 and cleaved caspases-3 1:1000 and subsequently with horseradish peroxidase-conjugated anti-rabbit or anti-mouse secondary antibody (1:3000; room temperature for 45 min). All membranes were stripped with Restore western blot stripping buffer (Pierce, # 21059, lot # FH71541), reprobed with an antibody specific to β-actin which was used as an internal standard. Peroxidase activity was visualized with the Super signal^® ^West Femto maximum sensitivity substrate (Pierce, Arlington Heights, IL, USA), according to the manufacturer's instructions. Signal was visualized using the Biochemi Imaging System (UVP, CA, USA). Densitometrical analyses were performed (protein of interest and β-actin) using the GelDoc 2000 and the Quantity One software (Bio-Rad, Mississauga, ON, Canada). Results are expressed as a ratio (protein of interest/β-actin) to correct for loading for each endometrial sample.

### Hoechst nuclear staining

Following TGF-β treatment, both floating and attached cells were resuspended in PBS containing Hoechst 33258 for 24 hours at 4°C. Hoechst nuclear staining was viewed and photographed using a Olympus BX60 fluorescence microscope and a Coolsnap-Pro CF digital Camera (Carsen Group, ON, Canada). Cells with typical apoptotic nuclear morphology (nuclear shrinkage, condensation and fragmentation) were identified and counted using randomly selected fields on numbered photographic slides, of which the "counter" was not aware of the treatment, so as to avoid experimental bias. A minimum of 200 cells per treatment group were counted in each experiment.

### Statistical analysis

Western analyses of cycling and pseudopregnant animals were repeated three to four times with samples collected from 3–4 different rats/day of pregnancy. Endometrial extracts from each rat were assessed individually. Western analyses of cultured decidual cells were repeated 4 times for each TGF-β dose (for each culture experiment, decidual cells were recovered from a pool of ten different ovariectomized/treated rats). Results subjected to statistical analyses were expressed as mean ± SEM. Data were subjected to one-way ANOVA (PRISM software version 4.0; GraphPad, San Diego, CA). Differences between experimental groups were determined by the Tukey's test.

## Results

### Regulation of TGF-β1, β2 and β3 in the rat endometrium during the estrous cycle

**Figure 1:**
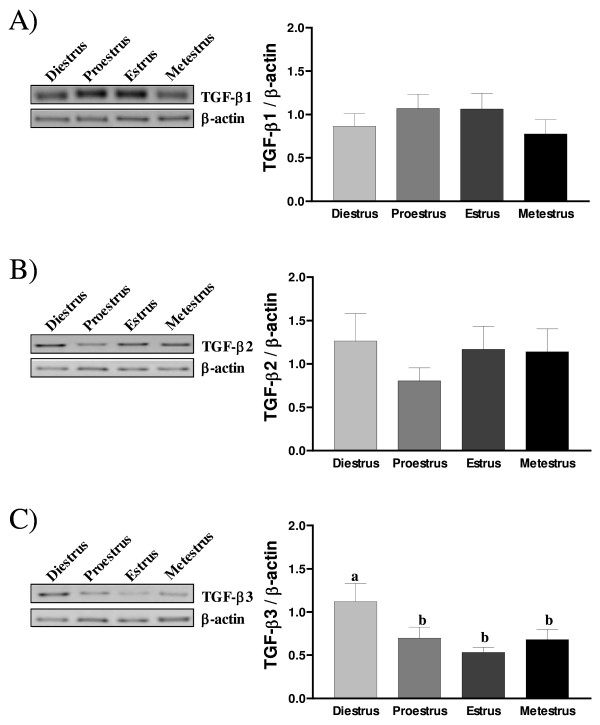
**TGF-β1 (A), TGF-β2 (B) and TGF-β3 (C) mRNAs abundance during the estrous cycle as demonstrated by RT-PCR analysis**. Total mRNA extracts from rat uteri were collected at each day of the estrous cycle. Data analyses were performed by the Quantity One software and are presented as a ratio (value/β-actin). β-actin blots shown were used as controls to correct for loading in each lane. Results represent the mean ± SEM of four independent experiments (four different rats for each day of the estrous cycle). Columns with different letters are significantly different from each other (P < 0.05).

To evaluate gene expression of TGF-β isoforms in rat endometrium during the estrous cycle, rat uteri where collected at each stage of the estrous cycle, total endometrial RNA was extracted and RT-PCR analysis was carried out using specific primers derived from rat DNA sequences. Our results showed that all three TGF-β isoforms were expressed throughout the estrous cycle. TGF-β 1 transcript levels tended to be higher at proestrus and estrus (Fig. 1A). TGF-β2 mRNA levels was similar at all days of estrous cycle, except at proestrus where it tended to decrease (Fig. 1B), and TGF-β3 mRNA levels were significantly increased (p < 0.05) on diestrus compared to other stages of the cycle (Fig, 1C).

**Figure 2:**
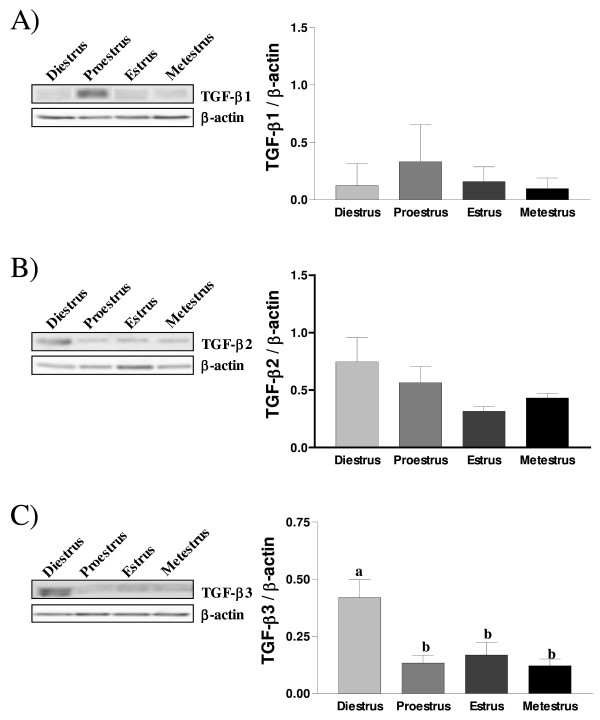
**TGF-β1 (A), TGF-β2 (B) and TGF-β3 (C) protein expression during the estrous cycle as determined by Western blot analysis**. Total proteins extracts from rat uteri were collected at each days of the estrous cycle. β-actin blots shown were used as controls to correct for loading in each lane. Blots shown are from one representative experiment. Graphics represent Western blot densitometrical analysis. Data represent the mean ± SEM of four independent experiments (four different rats for each day of the estrous cycle). Columns with different letters are significantly different from each other (P < 0.05).

To characterize TGF-β protein expression in rat endometrium during the estrous cycle, Western Blot and immunohistochemical analysis were performed on lysate and uterine sections, respectively, of reproductive cycling rats. Western blot analyses confirm that TGF-β isoforms are present during the estrous cycle. TGF-β1 protein levels tended to increase, although not significantly, during proestrus (Fig. 2A) as we observed with RT-PCR studies. The expression of TGF-β2 proteins were increased during diestrus but decreased at estrus (Fig. 2B). TGF-β3 proteins were expressed in a similar pattern as observed with mRNA expression; they were significantly increased during diestrus (Fig. 2C), suggesting a possible role for this isoform at diestrus.

**Figure 3:**
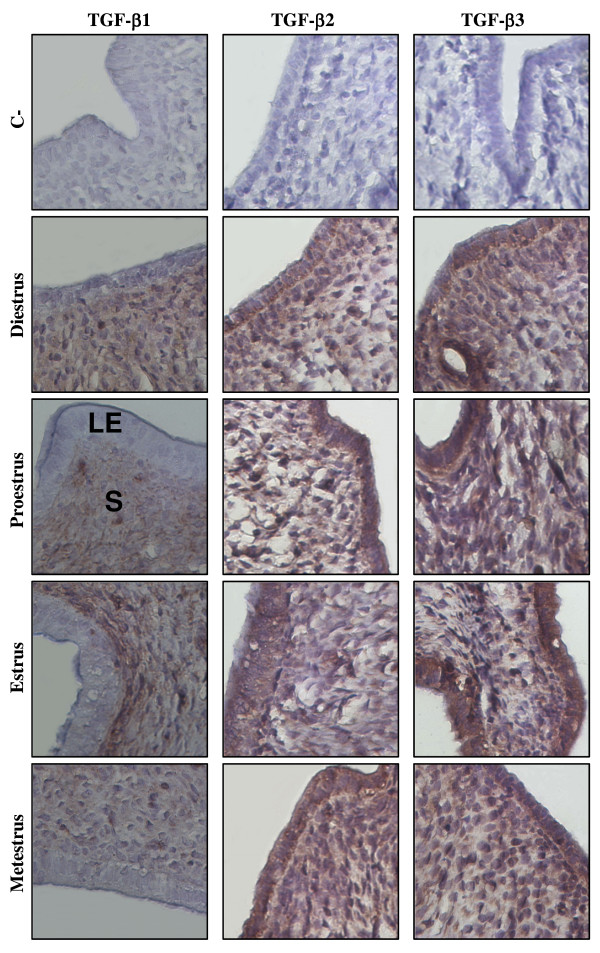
**Immunohistochemistry of TGF-β1, TGF-β2 and TGF-β3 in rat endometrium during the estrous cycle**. IHC shown are from one representative experiment and were repeated four times using four different uterine sections from four different rats per day of the estrous cycle. Representative days of estrous cycle are presented (diestrus; proestrus; estrus; metestrus). C-: negative control. LE: luminal epithelium; S: stroma. Magnification: 400×.

As TGF-β2 and TGF-β3 seem to be regulated in a similar pattern, we have carried out immunohistochemical analysis to determine their localization into the non-pregnant endometrium (Fig. 3). TGF-β2 and TGF-β3 were found both in luminal and glandular epithelial and stromal cells at all days of the reproductive cycle. However, it is interesting to observe that TGF-β1 is also found at each days of the estrous cycle but only in stromal cells (Fig. 3) suggesting that the action of TGF-β1 might be limited to the stromal compartment.

### Regulation of TGF-βs and apoptosis in the rat endometrium during pseudopregnancy

**Figure 4:**
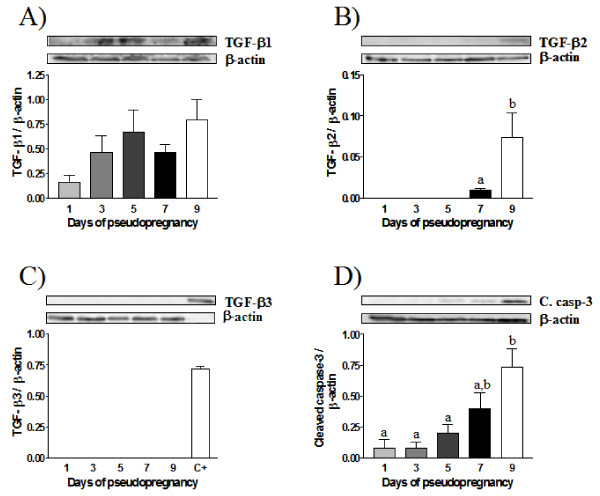
**TGF-β1 (A), TGF-β2 (B), TGF-β3 (C) and Cleaved Caspase-3 (D) proteins expression during the pseudopregnancy as determined by Western blot analysis**. Total proteins extracts were collected from uteri at each days of the pseudopregnancy. β-actin blots shown were used as controls to correct for loading in each lane. Blots shown are from one representative experiment. Graphics represent Western blot densitometrical analysis. Data represent the mean ± SEM of four independent experiments (four different rats for each day of the pseudopregnancy). Columns with different letters are significantly different from each other (P < 0.05). C+: positive control for TGF-β3.

In order to determine if the expression of TGF-β isoforms in the endometrium during pregnancy is dependant on the presence of a conceptus, we performed western blot analysis on endometrial protein extracts from a model of pseudopregnant rats. TGF-β1 expression was detected at all days of pseudopregnancy without significant difference and levels tended to increase on days 5 (implantation) and was maximal at day 9 (Fig. 4A). TGF-β2 was present only from day 7 of pseudopregnancy where its expression increased strongly on day 9 (Fig. 4B). However, TGF-β3 was not expressed during pseudopregnancy (Fig. 4C) suggesting that it may not be regulated and involved during early pregnancy.

It has been shown that apoptosis of epithelial endometrial cells is required for embryo implantation [13]. To determine the presence of apoptosis in the endometrium during pseudopregnancy, we have measured the levels of cleaved caspase-3, a well known executioner of apoptosis [41]. Our results show that cleaved caspase-3 is increased from day 5, day at which embryo implantation occurs, to day 9 of pseudopregnancy (Fig. 4D), indicating that apoptosis can occur during the implantation period in the absence of embryonic factors.

### Effect of TGF-β2 and TGF-β3 on P-Akt and XIAP protein levels in decidual cells

**Figure 5:**
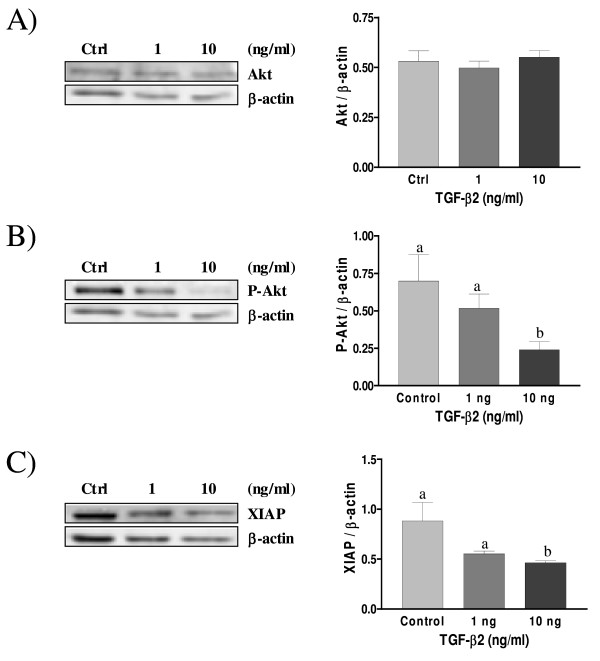
**Expression of Akt (A), Phospho-Akt (B) and XIAP (C) in cultured rat decidual cells *in vitro *in response to TGF-β2 (ng/mL) as demonstrated by Western blot analysis**. β-actin blots shown were used as controls to correct for loading in each lane. Blots shown are from one representative experiment. Graphics represent Western blot densitometrical analysis. Data represent the mean ± SEM of four independent experiments. Columns with different letters are significantly different from each other (P < 0.05).

**Figure 6:**
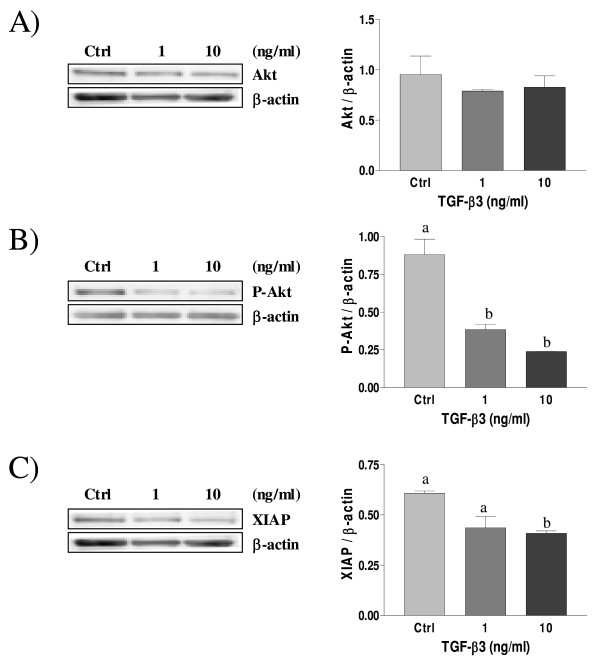
**Expression of Akt (A), Phospho-Akt (B) and XIAP (C) in cultured rat decidual cells *in vitro *in response to TGF-β3 (ng/mL) as demonstrated by Western blot analysis**. β-actin blots shown were used as controls to correct for loading in each lane. Blots shown are from one representative experiment. Graphics represent Western blot densitometrical analysis. Data represent the mean ± SEM of four independent experiments. Columns with different letters are significantly different from each other (P < 0.05).

We have previously shown that TGF-β1 down-regulates p-Akt (phosphorylated/activated form of the cell survival factor Akt) and XIAP (x-linked inhibitor of apoptosis) levels in rat decidual cells [38]. To determine the effect of TGF-β2 and TGF-β3 isoforms on the expression of cell survival protein, such as Akt and XIAP, and to further characterize the relationship between TGF-β and PI3-K/Akt pathways in decidual cells, Western blot analysis was performed on cultured decidual stromal cells treated with different dose of TGF-β2 and TGF-β3. The results showed that total Akt protein level is not influenced by TGF-β2 and TGF-β3 treatments and no significant variation was observed (Fig. 5A and 6A respectively). However, phosphorylated Akt (p-Akt), the active form of Akt, was decreased in a dose-dependant manner in response to TGF-β2 and TGF-β3 treatments (Fig. 5B and 6B respectively), suggesting an interaction between TGF-β isoforms and PI3-K/Akt pathway in these cells. TGF-β has been reported to regulate XIAP levels in several cell types such as in endometrial carcinoma cells [33], and we investigated whether down-regulation of Akt survival pathway activity in rat decidual cells by TGF-β2 and TGF-β3 was accompanied by a decrease in the levels of anti-apoptotic XIAP protein. Similar to TGF-β1, which we previously shown to reduce XIAP levels in rat cultured decidual cells [38], we found that XIAP levels were also reduced in a dose-dependant manner in response to TGF-β2 and TGF-β3 treatments (Fig. 5C and 6C respectively).

### TGF-β3 activates Smad2 in rat decidual cells

**Figure 7:**
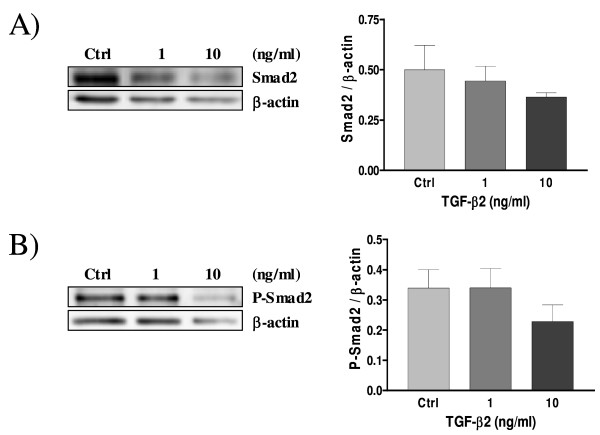
**Expression of Smad2 (A) and Phospho-Smad2 (B) in cultured rat decidual cells *in vitro *in response to TGF-β2 (ng/ml) as demonstrated by Western blot analysis**. β-actin blots shown were used as controls to correct for loading in each lane. Blots shown are from one representative experiment. Graphics represent Western blot densitometrical analysis. Data represent the mean ± SEM of four independent experiments.

**Figure 8:**
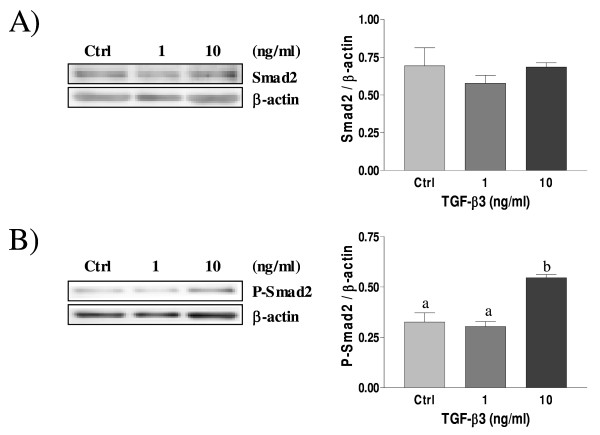
**Expression of Smad2 (A) and Phospho-Smad2 (B) in cultured rat decidual cells *in vitro *in response to TGF-β3 (ng/ml) as demonstrated by Western blot analysis**. β-actin blots shown were used as controls to correct for loading in each lane. Blots shown are from one representative experiment. Graphics represent Western blot densitometrical analysis. Data represent the mean ± SEM of four independent experiments. Columns with different letters are significantly different from each other (P < 0.05).

Smads proteins are known so far to be the TGF-β type I receptor substrates that elicit signalling function in response to TGF-β[25]. Western blot analysis was carried out to determine the intracellular mechanism of TGF-β isoforms action in rat decidual cells, and we specifically investigated Smad signalling components. Figure 7 show that there is no significant difference in the expression of Smad2 protein in response to TGF-β2 (Fig. 7A) and TGF-β3 (Fig. 8A) treatments. Phospho-Smad2, the activated form of Smad2 protein, was not influenced by TGF-β2 (Fig. 7B), however it was significantly increased in response to TGF-β3 treatment (Fig. 8B), indicating that TGF-β3 triggers Smad signalling in rat decidual cells, notably involving Smad2.

### TGF-β2 and β3 isoforms effect on decidual endometrial stromal cell fate

**Figure 9:**
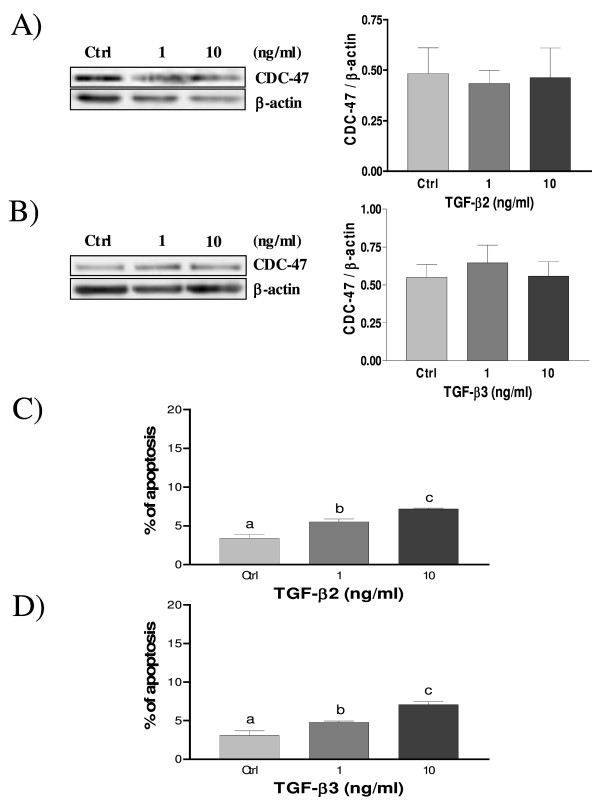
**Expression of CDC-47 in cultured rat decidual cells *in vitro *in response to TGF-β2 (A) and TGF-β3 (B) (ng/ml) as demonstrated by Western blot analysis**. β-actin blots shown were used as controls to correct for loading in each lane. Blots shown are from one representative experiment. Graphics represent Western blot densitometrical analysis. C) and D) effect of TGF-β2 and TGF-β3 (ng/ml) on cell survival in cultured rat decidual cells as demonstrated by Hoechst nuclear staining. Data represent the mean ± SEM of four independent experiments. Columns with different letters are significantly different from each other (P < 0.05).

Transforming growth factor-beta isoforms are multifunctional proteins involved in many cellular processes like cell proliferation, differentiation and apoptosis and these actions are depending of the tissue [17]. We have previously determined that TGF-β1 decreased decidual cell proliferation [38]. We have performed Western blot analysis using CDC-47, a cell proliferation marker, to determine the proliferative action of the TGF-β2 and TGF-β3 isoforms on decidual stromal cells. Results show that CDC-47 levels were not significantly modulated in response to TGF-β2 and TGF-β3 treatments (Fig. 9A and 9B) suggesting that TGF-β2 and TGF-β3 do not influence decidual cell growth.

It was already demonstrated that TGF-β induced apoptosis in rat endometrium [8]. To evaluate whether TGF-β2 and TGF-β3 induce apoptosis in rat decidual cells, we have carried out Hoechst nuclear staining on cultured decidual cells treated with different doses of TGF-β2 and TGF-β3. Our results demonstrate that both TGF-β isoforms induced cell death in a dose-dependent manner (Fig. 9C and 9D), however the increase of cell death was lower compared to TGF-β1-induced cell death in the same type of cells [38].

## Discussion

Although TGF-β isoforms have been shown to be regulated in the rat endometrium during pregnancy [38], little is known about their expression during the estrous cycle and their action on endometrial decidual cell regression. The aim of this study was to investigate and characterize the expression of TGF-β isoforms in the rat endometrium throughout the estrous cycle and during pseudopregnancy and to determine the specific effect of TGF-β2 and TGF-β3 *in vitro *on rat endometrial decidual cell fate. The present results revealed that TGF-β isoforms are differently regulated during the estrous cycle and pseudopregnancy and they mediate apoptosis of decidual cells.

During the estrous cycle, the uterine endometrial cells undergo proliferation, differentiation and apoptosis in response to the steroids hormones, estrogen and progesterone, to create a suitable environment for blastocyst implantation [42]. Moreover, a complex, precise, and highly interactive array of biological communication pathways involving the action of steroids in concert with local growth factors and cytokines is required to orchestrate the controlled growth and differentiation of the endometrial cells in the uterus [43]. TGF-β isoforms are known to be implicated in diverse physiological processes and they regulate cell proliferation, differentiation and apoptosis. Since all of these events take place in the endometrium during the estrous cycle [44], TGF-β isoforms must be an important growth factor involved during the reproductive cycle. We have thus characterized the expression of TGF-β in the endometrium of cycling rats. Our results show that the three mammalian TGF-β isoforms are present during all stage of the estrous cycle, which is consistent with a previous study that showed, using immunohistochemical analysis that TGF-β isoforms are expressed in rat endometrium during the cycle [34]. It is known that apoptosis is present in rat endometrium during the estrous cycle [9] and that TGF-β induce apoptosis in rat endometrial stromal cells [8]. In the present study, TGF-β1 expression tended to be increased during proestrus even though no significant difference was observed and it was localized only in stromal cells suggesting a role for this isoform in the remodelling of stromal tissue. Additionally, we have demonstrated that TGF-β2 and TGF-β3 expression was increased during diestrus, which is in accordance with the results of Chegini et al [34], and they were localized in both epithelial and stromal cells suggesting that these two isoforms might be necessary to induce apoptosis in stromal cells during diestrus stage. This result is in accordance with a previous study showing that apoptosis was increased in stromal cells during diestrus in the mouse [4]. However, we and other have previously shown the presence of apoptosis in rat uterine epithelial cells at estrus [10,45], but we found no increase in TGF-β levels during estrus suggesting that TGF-β2 and TGF-β3 might be involved in cellular processes other than apoptosis in rat epithelial endometrial cells during the estrous cycle. Alternatively, downstream components of TGF-β receptor could be differently regulated in distinct cells of the same tissue resulting in increased sensitivity to TGF-β induced apoptosis in rat stromal endometrial cells during diestrus.

The high levels of TGF-β2 and TGF-β3 at diestrus are correlated with elevated levels of serum progesterone at this time. Indeed, Dharma et al [4] showed in the uterine endometrium of the mouse that serum progesterone levels were high during metestrus, the day before diestrus. Moreover, studies showed that estrogen induces uterine epithelial cell proliferation and estrogen withdrawal results in cell death [10,46], which might suggests that epithelial cell apoptosis in the uterine endometrium is regulated by estrogen while stromal cell apoptosis might be regulated by progesterone [4]. Since some evidence suggests that steroid action stimulates the local production of growth factors and their receptors [47,48] and that growth factors have been implicated in mediating the action of steroid hormones [47-49], TGF-β might play a similar role in the induction of progesterone-mediated apoptosis during the estrous cycle. A recent study showed that Smad2, a component of the TGF-β intracellular signaling pathway, was highly expressed in the luminal and glandular epithelium at diestrus and proestrus while it was decreased at estrus and metestrus [50] suggesting that TGF-β might play an important role through Smad2 in tissue remodelling in the cycling rat endometrium.

Other roles than apoptosis induction can be suggested for TGF-β during the estrous cycle. It has been shown that TGF-β is a potent chemoattractant for macrophages [19] and monocytes [20], suggesting a role for this growth factor in the elimination of dead cells before the next cycle (the next ovulation) and in wound repair. Furthermore, it has been shown that matrix metalloproteinase (MMP), an important class of proteases involved in endometrial remodelling, was present in the rat endometrium during the estrous cycle; the presence of MMP-3, MMP-9 and MMP-13 (primary source of collagenase activity first associated with uterine involution) has been demonstrated in the stromal cells of non-pregnant rat endometrium, while epithelial cell-specific MMP-7 (first MMP protein linked to matrix remodelling in the rodent uterus) has also been demonstrated in the non-pregnant rat uterus [43]. A previous study showed that MMP-7 mRNA levels were dependant on the estrous cycle and it was 3 to 4 fold higher at estrus and diestrus compared to metestrus [51]. Both TGF-β and MMPs are recognized to have an action on tissue remodelling, and TGF-β might act through the regulation of MMP activity. In fact, it has been shown that TGF-β plays an important role in the regulation of MMP production in the endometrium during the menstrual cycle in women [43,52]. However, little is known about the interaction between TGF-β and matrix metalloproteinase in the remodelling uterus in cycling rats and further investigations have to be carried out.

During early pregnancy, uterine endometrium is alternatively remodelled by sex steroid and growth factors to allow embryo implantation and then decidual regression near the end of pregnancy. Apoptosis has been shown to occur during these two important processes [5,8,13]. Several growth factors are produced and secreted by both conceptus and maternal uterine tissues in embryo-endometrial interactions during implantation and decidual regression. We have previously demonstrated that TGF-β isoforms are present and expressed differently in the rat endometrium during pregnancy [38]. Since the induction of TGF-α gene expression has been shown to be independent of the conceptus [53], we hypothesized that it might be similar for TGF-β. Using the rat pseudopregnancy model, which involves pregnancy-like remodelling of the endometrium following exposure to steroid hormones, we showed that TGF-β isoforms are differently expressed and regulated in rat endometrial cells during pseudopregnancy in a pattern similar to pregnant uterus [38]. Notably, we have demonstrated that TGF-β1 was present in all days of pseudopregnancy but its expression was increased on day 5, day at which embryo implantation normally occurs, and was maximal on day 9 in a similar pattern as compared to normal pregnancy [38], strengthening our hypothesis that TGF-β1 might be involved in the regulation of apoptosis during embryo implantation and decidual regression, without the need for signals from the embryo. TGF-β2 levels were shown to be elevated only on day 9 of pseudopregnancy, and TGF-β3 has not been detected during pseudopregnancy, similarly to what was observed during normal pregnancy [38], confirming that this isoform does not play a crucial role during early pregnancy. This result is also supported by a previous work demonstrating that TGF-β3 was absent during early pregnancy in the mouse [32]. Altogether, our results indicate that TGF-β isoforms expression is independent of the conceptus (embryonic factors) and it might be regulated by steroid hormones. Moreover, this differential expression of TGF-β isoforms in the uterus suggests distinctive functions of these growth factors during early pregnancy.

Our results also show that apoptosis is induced during pseudopregnancy. We have demonstrated that cleaved caspase-3, a well known executioner of apoptosis [41], was present in endometrium days 5 to 9 of pseudopregnancy. Its levels were increased in a similar pattern than what was observed during pregnancy [38] from day 5, day at which embryo implantation occurs, to day 9 of pseudopregnancy, indicating that the caspases pathway activation might be an important mechanism of cell death involved in implantation and decidual regression.

Akt kinase is a key regulator of cell fate in numerous cell types, including endometrial cells [10]; since TGF-β signalling pathway component Smad3 can directly interact with Akt [53], we hypothesized that a crosstalk between TGF-β and PI3-K/Akt signalling pathways could occur in decidual cells which could dictate cell fate during pregnancy. It is already known that TGF-β1 induce apoptosis in human [37] and rat [8] endometrial stromal cells, and we have demonstrated in a previous work that TGF-β1 regulates the activity of PI3-K/Akt pathway, inducing a decrease of Akt phosphorylation through a mechanism involving Smad2 activation [38]. The present study shows that, similar to TGF-β1 [38], TGF-β3 induces the phosphorylation/activation of Smad2 and decreases Akt phosphorylation/activation in a dose-dependant manner, and that TGF-β2 also decreases Akt phosphorylation, although likely in a Smad2-independent fashion. It is possible that upon binding to its ligand TGF-β2, TGF-β receptor signals through Smad3 rather than Smad2. Indeed, recent studies showed that Akt interacts directly with Smad3 to regulate the sensitivity to TGF-β-induced apoptosis [54,55], and we hypothesize that TGF-β2 might decrease P-Akt levels through Smad3 activation in rat decidual cells. Moreover, the reduction of Akt phosphorylation in response to TGF-β2 and TGF-β3 suggest that inhibition of Akt activity might be an important mechanism by which these TGF-β isoforms induce apoptosis in rat decidual cells.

Recent studies have shown the important interaction between Akt and XIAP, a well known inhibitor of caspases. It was shown that Akt phosphorylation is regulated by XIAP in human ovarian epithelial cancer cells [56] and in rat granulosa cells [57] and that phosphorylated Akt is affected by the presence of cIAP-1 in endometrial cancer cells [58]. More recently, we have shown that XIAP is an E3 ubiquitin ligase for PTEN (phosphatase and tensin homolog deleted on chromosome ten, a negative regulator of Akt) and that degradation of PTEN through the proteasome lead to the activation/phosphorylation of Akt [59]. We have also previously shown that TGF-β1 reduced XIAP protein levels in rat decidual cells, suggesting the presence of a functional link between these two proteins [38]. Here, we show that, in response to TGF-β2 and TGF-β3, XIAP levels are decreased in a dose-dependant manner. Studies have shown that XIAP can act as a cofactor in the TGF-β signalling pathway [22,60]. Downregulation of the anti-apoptotic factor XIAP is accompanied by a modest but significant induction of apoptosis in rat decidual cells exposed to TGF-β2 and TGF-β3, suggesting that XIAP can inhibit apoptotic mechanisms in rat decidual cells.

A study showed that TGF-β1 and TGF-β2 treatments on cultured stromal cells induce apoptosis [8]. In this paper, we show that TGF-β2 and TGF-β3 have no effect on the expression of CDC-47, a proliferation cell marker, suggesting that these isoforms do not modulate cell proliferation of endometrial decidual cells although they weakly induce cell death in a dose-dependant manner as demonstrated by Hoechst nuclear staining. This is a surprising observation, since increased cell death is usually accompanied by a decrease in cellular proliferation marker levels. It is possible that other proliferation markers different than CDC47 would be more relevant to decidual cells, such as the proliferating cell nuclear antigen (PCNA) [61]. Moreover, the weak increase of apoptosis observed with Hoechst staining might be accompanied by other types of cell death such as autophagy, mitotic catastrophe or senescence. Indeed, TGF-β has been showed to induce senescence phenotype or to have a cytostatic action (cell cycle arrest) on mammalian tissues [62].

Further investigation is thus needed to better understand the interaction between TGF-βs and PI3-K/Akt pathway and to determine how other factors that might be involved in the regulation of TGF-β-mediated apoptosis of decidual cells.

## Conclusion

Taken together, these results show that the three TGF-β isoforms are expressed and differently localized in the epithelial and stromal cells during the estrous cycle, and that they are differently regulated during pseudopregnancy in a steroid hormone-dependent manner. Moreover, this study shows that TGF-β signalling plays an important role in the remodelling of rat endometrium by modulating the activity of PI 3-K/Akt survival pathway, down-regulating the anti-apoptotic XIAP levels and inducing apoptosis in decidual cells. Future experiments will aim at better characterize the underlying mechanisms such as the possible interaction between TGF-β2 and Smad proteins in the TGF-β-mediated apoptosis and the mechanism by which TGF-β and Smads transducer proteins interact with PI 3-K/PTEN/Akt survival pathway to induce apoptosis. Additional investigations will allow us to determine the presence of other type of programmed cell death such as autophagy, senescence and mitotic catastrophe and how they are involved in the rat endometrial decidual cell death.

## Competing interests

The authors declare that they have no competing interests.

## Authors' contributions

PLC performed the experiments and drafted the manuscript. GFF participated in the animal studies. CS helped with the experiments. VL helped with cell cultures and animal experiments. EA designed the study and finalized writing of the manuscript.

## References

[B1] Graham JD, Clarke CL (1997). Physiological action of progesterone in target tissues. Endocr Rev.

[B2] Martin L, Das RM, Finn CA (1973). The inhibition by progesterone of uterine epithelial proliferation in the mouse. J Endocrinol.

[B3] Mendoza-Rodriguez CA, Monroy-Mendoza MG, Morimoto S, Cerbon MA (2003). Pro-apoptotic signals of the bcl-2 gene family in the rat uterus occur in the night before the day of estrus and precedes ovulation. Mol Cell Endocrinol.

[B4] Dharma SJ, Kholkute SD, Nandedkar TD (2001). Apoptosis in endometrium of mouse during estrous cycle. Indian J Exp Biol.

[B5] Abrahamsohn PA, Zorn TM (1993). Implantation and decidualization in rodents. J Exp Zool.

[B6] Nuttall RK, Kennedy TG (2000). Epidermal growth factor and basic fibroblast growth factor increase the production of matrix metalloproteinases during in vitro decidualization of rat endometrial stromal cells. Endocrinology.

[B7] Welsh AO (1993). Uterine cell death during implantation and early placentation. Microsc Res Tech.

[B8] Moulton BC (1994). Transforming growth factor-beta stimulates endometrial stromal apoptosis in vitro. Endocrinology.

[B9] Sato T, Fukazawa Y, Kojima H, Enari M, Iguchi T, Ohta Y (1997). Apoptotic cell death during the estrous cycle in the rat uterus and vagina. Anat Rec.

[B10] Dery MC, Leblanc V, Shooner C, Asselin E (2003). Regulation of Akt expression and phosphorylation by 17beta-estradiol in the rat uterus during estrous cycle. Reprod Biol Endocrinol.

[B11] Leblanc V, Dery MC, Shooner C, Asselin E (2003). Opposite regulation of XIAP and Smac/DIABLO in the rat endometrium in response to 17beta-estradiol at estrus. Reprod Biol Endocrinol.

[B12] Gu Y, Jow GM, Moulton BC, Lee C, Sensibar JA, Park-Sarge OK, Chen TJ, Gibori G (1994). Apoptosis in decidual tissue regression and reorganization. Endocrinology.

[B13] Pampfer S, Donnay I (1999). Apoptosis at the time of embryo implantation in mouse and rat. Cell Death Differ.

[B14] Moses HL, Branum EL, Proper JA, Robinson RA (1981). Transforming growth factor production by chemically transformed cells. Cancer Res.

[B15] Roberts AB, Anzano MA, Lamb LC, Smith JM, Sporn MB (1981). New class of transforming growth factors potentiated by epidermal growth factor: isolation from non-neoplastic tissues. Proc Natl Acad Sci USA.

[B16] Roelen BA, Lin HY, Knezevic V, Freund E, Mummery CL (1994). Expression of TGF-beta s and their receptors during implantation and organogenesis of the mouse embryo. Dev Biol.

[B17] Massague J (1990). The transforming growth factor-beta family. Annu Rev Cell Biol.

[B18] Massague J (1998). TGF-beta signal transduction. Annu Rev Biochem.

[B19] Barnard JA, Lyons RM, Moses HL (1990). The cell biology of transforming growth factor beta. Biochim Biophys Acta.

[B20] Attisano L, Wrana JL, Lopez-Casillas F, Massague J (1994). TGF-beta receptors and actions. Biochim Biophys Acta.

[B21] Hu PP, Datto MB, Wang XF (1998). Molecular mechanisms of transforming growth factor-beta signaling. Endocr Rev.

[B22] Herrera B, Fernandez M, Benito M, Fabregat I (2002). cIAP-1, but not XIAP, is cleaved by caspases during the apoptosis induced by TGF-beta in fetal rat hepatocytes. FEBS Lett.

[B23] Inman GJ, Allday MJ (2000). Apoptosis induced by TGF-β1 in Burkitt's lymphoma cells is caspase 8 dependent but is death receptor independent. J Immunol.

[B24] Govinden R, Bhoola KD (2003). Genealogy, expression, and cellular function of transforming growth factor-beta. Pharmacol Ther.

[B25] Lutz M, Knaus P (2002). Integration of the TGF-β pathway into the cellular signalling network. Cell Signal.

[B26] Hata A, Shi Y, Massague J (1998). TGF-beta signaling and cancer: structural and functional consequences of mutations in Smads. Mol Med Today.

[B27] Yingling JM, Wang XF, Bassing CH (1995). Signaling by the transforming growth factor-beta receptors. Biochim Biophys Acta.

[B28] Pelton RW, Saxena B, Jones M, Moses HL, Gold LI (1991). Immunohistochemical localization of TGF beta 1, TGF beta 2, and TGF beta 3 in the mouse embryo: expression patterns suggest multiple roles during embryonic development. J Cell Biol.

[B29] Gupta A, Ing NH, Bazer FW, Bustamante LS, Jaeger LA (1998). Beta transforming growth factors (TGFss) at the porcine conceptusmaternal interface. Part I: expression of TGFbeta1, TGFbeta2, and TGFbeta3 messenger ribonucleic acids. Biol Reprod.

[B30] Gupta A, Dekaney CM, Bazer FW, Madrigal MM, Jaeger LA (1998). Beta transforming growth factors (TGFbeta) at the porcine conceptus-maternal interface. Part II: uterine TGFbeta bioactivity and expression of immunoreactive TGFbetas (TGFbeta1, TGFbeta2, and TGFbeta3) and their receptors (type I and type II). Biol Reprod.

[B31] Munson L, Wilhite A, Boltz VF, Wilkinson JE (1996). Transforming growth factor beta in bovine placentas. Biol Reprod.

[B32] Das SK, Flanders KC, Andrews GK, Dey SK (1992). Expression of transforming growth factor-beta isoforms (beta 2 and beta 3) in the mouse uterus: analysis of the periimplantation period and effects of ovarian steroids. Endocrinology.

[B33] Van Themsche C, Mathieu I, Parent S, Asselin E (2007). Transforming growth factor-beta3 increases the invasiveness of endometrial carcinoma cells through phosphatidylinositol 3-kinase-dependent upregulation of X-linked inhibitor of apoptosis and protein kinase C-dependent induction of matrix metalloproteinase. J Biol Chem.

[B34] Chegini N, Gold LI, Williams RS (1994). Localization of transforming growth factor beta isoforms TGF-beta 1, TGF-beta 2, and TGF-beta 3 in surgically induced endometriosis in the rat. Obstet Gyneco.

[B35] Tamada H, McMaster MT, Flanders KC, Andrews GK, Dey SK (1990). Cell type-specific expression of transforming growth factor-beta1 in the mouse uterus during the periimplantation period. Mol Endocrinol.

[B36] Manova K, Paynton BV, Bachvarova RF (1992). Expression of activins and TGF beta 1 and beta 2 RNAs in early postimplantation mouse embryos and uterine decidua. Mech Dev.

[B37] Chatzaki E, Kouimtzoglou E, Margioris AN, Gravanis A (2003). Transforming growth factor beta1 exerts an autocrine regulatory effect on human endometrial stromal cell apoptosis, involving the FasL and Bcl-2 apoptotic pathways. Mol Hum Reprod.

[B38] Shooner C, Caron PL, Frechette-Frigon G, Leblanc V, Dery MC, Asselin E (2005). TGF-beta expression during rat pregnancy and activity on decidual cell survival. Reprod Biol Endocrinol.

[B39] Kennedy TG, Ross HE (1997). Temporal- and hormone-dependent changes in uterine sensitization for the decidual cell reaction and decidualization in vitro of rat endometrial stromal cells. J Reprod Fertil.

[B40] Kennedy TG, Ross HE (1993). Effect of prostaglandin E2 on rate of decidualization in rats. Prostaglandins.

[B41] Cohen GM (1997). Caspases: the executioners of apoptosis. Biochem J.

[B42] Erickson GF, Fuqua L, Shimasaki S (2004). Analysis of spatial and temporal expression patterns of bone morphogenetic protein family members in the rat uterus over the estrous cycle. J Endocrinol.

[B43] Curry TE, Osteen KG (2003). The matrix metalloproteinase system: changes, regulation, and impact throughout the ovarian and uterine reproductive cycle. Endocr Rev.

[B44] Godkin JD, Dore JJ (1998). Transforming growth factor beta and the endometrium. Rev Reprod.

[B45] Lai MD, Lee LR, Cheng KS, Wing LY (2000). Expression of proliferating cell nuclear antigen in luminal epithelium during the growth and regression of rat uterus. J Endocrinol.

[B46] Finn CA, Publicover M (1981). Hormonal control of cell death in the luminal epithelium of the mouse uterus. J Endocrinol.

[B47] Giudice LC (1994). Growth factors and growth modulators in human uterine endometrium: their potential relevance to reproductive medicine. Fertil Steril.

[B48] Ignar-Trowbridge DM, Nelson KG, Bidwell MC, Curtis SW, Washburn TF, McLachlan JA, Korach KS (1992). Coupling of dual signaling pathways: epidermal growth factor action involves the estrogen receptor. Proc Natl Acad Sci USA.

[B49] Piva M, Flieger O, Rider V (1996). Growth factor control of cultured rat uterine stromal cell proliferation is progesterone dependent. Biol Reprod.

[B50] Lin HY, Wang HM, Li QL, Liu DL, Zhang X, Liu GY, Qian D, Zhu C (2004). Expression of Smad2 and Smad4, transforming growth factor-beta signal transducers in rat endometrium during the estrous cycle, pre-, and peri-implantation. Anim Reprod Sci.

[B51] Wolf K, Sandner P, Kurtz A, Moll W (1996). Messenger ribonucleic acid levels of collagenase (MMP-13) and matrilysin (MMP-7) in virgin, pregnant, and postpartum uterus and cervix of rat. Endocrinology.

[B52] Bruner KL, Rodgers WH, Gold LI, Korc M, Hargrove JT, Matrisian LM, Osteen KG (1995). Transforming growth factor beta mediates the progesterone suppression of an epithelial metalloproteinase by adjacent stroma in the human endometrium. Proc Natl Acad Sci USA.

[B53] Bonvissuto AC, Lala PK, Kennedy TG, Nygard K, Lee DC, Han VK (1992). Induction of transforming growth factor-alpha gene expression in rat decidua is independent of the conceptus. Biol Reprod.

[B54] Remy I, Montmarquette A, Michnick SW (2004). PKB/Akt modulates TGF-beta signalling through a direct interaction with Smad3. Nat Cell Biol.

[B55] Conery AR, Cao Y, Thompson EA, Townsend CMJ, Ko TC, Luo K (2004). Akt interacts directly with Smad3 to regulate the sensitivity to TGF-beta induced apoptosis. Nat Cell Biol.

[B56] Asselin E, Mills GB, Tsang BK (2001). XIAP regulates Akt activity and caspase-3-dependent cleavage during cisplatin-induced apoptosis in human ovarian epithelial cancer cells. Cancer Res.

[B57] Asselin E, Wang Y, Tsang BK (2001). X-linked inhibitor of apoptosis protein activates the phosphatidylinositol 3-kinase/Akt pathway in rat granulosa cells during follicular development. Endocrinology.

[B58] Gagnon V, St-Germain ME, Parent S, Asselin E (2003). Akt activity in endometrial cancer cells: regulation of cell survival through cIAP-1. Int J Oncol.

[B59] Van Themsche C, Leblanc V, Parent S, Asselin E (2009). XIAP regulates PTEN ubiquitination, content and compartmentalization. J Biol Chem.

[B60] Birkey Reffey S, Wurthner JU, Parks WT, Roberts AB, Duckett CS (2001). X-linked inhibitor of apoptosis protein functions as a cofactor in transforming growth factor-beta signaling. J Biol Chem.

[B61] Ogle TF, George P, Dai D (1998). Progesterone and estrogen regulation of rat decidual cell expression of proliferating cell nuclear antigen. Biol Reprod.

[B62] Siegel PM, Massague J (2003). Cytostatic and apoptotic actions of TGF-β in homeostasis and cancer. Nat Rev Cancer.

